# What Could Be the Role of Antifungal Lock-Solutions? From Bench to Bedside

**DOI:** 10.3390/pathogens7010006

**Published:** 2018-01-06

**Authors:** Christine Imbert, Blandine Rammaert

**Affiliations:** 1 Laboratory of Ecology and Biology of Interactions, University of Poitiers, UMR CNRS 7267, F-86073 Poitiers, France; 2 Faculty of Medicine and Pharmacy, University of Poitiers, F-86073 Poitiers, France; blandine.rammaert.paltrie@univ-poitiers.fr; 3 CHU Poitiers, Infectious and Tropical Disease Unit, F-86073 Poitiers, France; 4 INSERM U1070, F-86073 Poitiers, France

**Keywords:** biofilm, antifungal agents, candidemia, catheter carrier patient

## Abstract

Candidemia related to the presence of a biofilm are often reported in patients with vascular catheters. Once they are mature, biofilms are persistent infectious reservoirs, and the yeasts dispersed from biofilms can cause infections. Sessile yeasts typically display increased levels of resistance to most antimicrobial agents and systemic treatments usually fail to eradicate previously formed fungal biofilms. In a curative strategy, antifungal lock therapy may help to sterilize catheters, with very high concentrations of antifungal agents, which are not compatible with systemic use. This strategy has been studied by several authors in in vitro and in vivo studies, and more rarely, in clinical settings for adult and paediatric patients. Our study aims to assess the efficacy of the antifungal solutions used for lock therapy and demonstrated by the different teams.

## 1. Introduction

*Candida albicans* belongs to human microflora and is a major opportunistic human pathogen. This species is responsible for a variety of diseases, ranging from oral thrush to candidemia, depending on patient status and fragility and/or immunosuppression level. Different factors contribute to the pathogenic potential of *C. albicans*. Although adherence ability and biofilm lifestyle are among the most important factors, we may also mention thigmotropism, yeast-to-hypha transition, secretion of hydrolases, and so on [1,2,3]. Adherence to an inert or living surface is the earliest phase required during the multi-step mechanism leading to formation of a biofilm. Biofilm has been defined as a structured community of microbial cells enclosed in a self-produced polymeric matrix [4]. In biofilms, sessile cells typically display increased levels of resistance to most antimicrobial agents and to host defence mechanisms, as well [5,6]. The ability of *C. albicans* to form biofilms associated with biotic surfaces, such as oral mucosa, and abiotic surfaces, such as implanted devices, can strongly impact human health. Importantly, biofilms are persistent infectious reservoirs of yeast cells dispersed from the biofilm that cause local and systemic infections [7]. Yeast cells dispersed from biofilms are not similar to regular planktonic yeast cells. In a recent study, Uppuluri and collaborators demonstrated that cells are likely to be dispersed primarily as yeast and that dispersion tends to occur continuously throughout biofilm development rather than being a massive event [8]. They also conclude that compared to their planktonic counterparts, dispersed cells display distinct phenotypic properties, including enhanced adherence, filamentation and pathogenicity in a murine model.

Ideally, a fully effective antimicrobial candidate treatment would kill the free microbial cells dispersed from the biofilm at the same time as the eradication of the biofilm itself. It would also be really advantageous if this ideal treatment totally removed the biofilm and made the catheter surface totally smooth so as to avoid or at least delay the formation of a new biofilm. It could be based on only one or a combination of molecules. In this way, the acute infection would be cured and relapse certainly delayed. Unfortunately, this ideal treatment candidate has yet to be discovered.

Candidemia are associated with reported mortality rates ranging from 30% to 60% and attributable mortality rates between 25% and 40%, *C. albicans* being the main species involved [9,10,11,12,13,14]. Outer and inner surfaces of vascular catheters, especially long-term ones, are particularly good substrates for *C. albicans* biofilm development, and are consequently at high risk of biofilm-related candidemia for patients. In case of a catheter-related bloodstream infection (CRBSI) caused by *Candida* spp., catheter removal is currently recommended. It has been widely shown that non-removal is associated with increased mortality and more prolonged candidemia [15,16,17,18,19,20,21,22,23,24]. This recommendation has certainly been given due to the insufficient activity of currently available systemic antifungal agents against *Candida* spp. biofilms. Many in vitro and in vivo studies have evaluated the ability of azoles, echinocandins and amphotericin B to eradicate a previously formed biofilm. They generally showed that azoles had poor activity against *C. albicans* biofilms. On the contrary, echinocandins and lipid formulations of amphotericin B generally demonstrated higher activity, even if total eradication was not obtained. Lipid formulations of amphotericin B appeared more effective than amphotericin B deoxycholate [5,6,9,25,26,27,28,29].

However, catheter replacement is not possible for all patients. For instance, in neonatology units extremely low birth weight infants are particularly at risk for CRBSI [30]. Candidemia occurs in approximately 10% of reported bloodstream infections in neonates [31]. The risk of CRBSI in neonatology unit has been shown to increase during the 2 weeks of catheter insertion [32]. Limited or no alternative intravenous access, particularly in children, leads to development of new approaches when catheters are infected with *Candida*. So, antifungal lock therapy may be helpful for these patients, in combination with conventional antifungal systemic therapy. Lock therapy involves instilling high concentrations of antimicrobial agents (usually from 100- to 1000-fold the minimal inhibitory concentration (MIC)) into the catheter lumen for extended periods of time [33,34]. This strategy is rather well-documented and defined for antiseptic and antibiotic approaches. However, the role of antifungal lock therapy against *Candida* is still not well-defined [15]. Biofilms may be located on both the outer and inner surfaces of vascular catheters. Of course, in case of an extra-luminal biofilm, antifungal lock would fail to eradicate biofilm.

The aim of the current article is to focus on the data available in the literature giving information about antifungal lock therapy approach in order to have better knowledge of this strategy. We will describe its efficacy and the conditions that would be the most appropriate in terms of antifungal agent, concentration, contact duration, and treatment frequency. There will be three parts successively focusing on in vitro, in vivo and clinical data. Only conventional antifungal agents will be included in this study. Many teams have developed in vitro models mimicking lock strategy to study the interest of the main available antifungal agents used as lock solutions. The main issue is the variability of the developed models that complicate comparative analysis of the results available in literature. Indeed, lock concentration, time and number of repeats, as well as biofilm substrates, maturation status of treated biofilms, and the method used to evaluate lock activity differ frequently according to the studies (Table 1). However, published results are generally in agreement and we will try below to summarize the main ideas regarding the efficacy of antifungal locks against *Candida* spp. biofilms.

## 2. Azole Lock Solutions

In most instances, all available data suggested that azole lock solutions fail to eradicate preformed *C. albicans* biofilms, regardless of test conditions. Öncü studied the efficacy of three azole (fluconazole, itraconazole and voriconazole) locks to eradicate a mature *C. albicans* biofilm, 5 days old, previously formed on silicone catheters, using only one strain. Fungal eradication was evaluated by determining the viable count. The author showed that no azole sterilized catheters, with more than 10^5^ CFU after the 7-day lock period compared to more than 10^6^ CFU on the non-treated catheters. Very high concentrations of major azole solutions failed to eradicate biofilms despite extended lock duration (24 h each) and repetition (for 7 days) [38]. Fiori et al. also tested fluconazole and voriconazole lock solutions, and included 20 clinical strains of *C. albicans* in their study; biofilms were formed on polystyrene surfaces (96-well microtiter-based method) but their maturation status was unclear [37]. The lowest azole concentrations at which a 50% decrease in biofilm metabolic activity (2,3-bis-(2-methoxy-4-nitro-5-sulfophenyl)-2*H*-tetrazolium-5-carboxanilide [XTT] method) was observed were always >128 µg/mL (fluconazole and voriconazole), confirming their inefficiency against *C. albicans* biofilms [37]. These lock solutions were also tested against numerous *C.* non-*albicans* biofilms (*C. glabrata*, *C. parapsilosis*, *C. tropicalis* and *C. krusei*). Again, the minimal concentrations reducing biofilms by half always exceeded >128 µg/mL [37]. Our team evaluated the efficacy of posaconazole lock solutions to eradicate 12 h-young and 5-day-old mature biofilms of *C. albicans* previously formed on silicone catheters. In this study posaconazole was tested at 10 mg/L corresponding to concentrations approximately 100–200 MIC and 16 strains of *Candida* sp. (ten of *C. albicans* and six of *C. glabrata*) were studied [26]. Posaconazole induced *C. albicans* biofilm inhibition ranging from 54.2% (young biofilms) to 57.6% (mature) 24 h after lock solution removal. Inhibition persisted over a 48 h post-lock period (inhibition ranging from 49.7% to 48.4% respectively). In this study posaconazole halved *C. albicans* biofilms regardless of their maturity level with a persistent effect. However, under the same test conditions posaconazole did not inhibit *C. glabrata* biofilms [26]. These results concurred with those reported by other teams who obtained no more than 40% inhibition of *C. albicans* biofilm tested at different maturation phases (24 h to 72 h-old biofilms) [42,43]. In their study Katragkou et al. tested voriconazole and posaconazole concentrations up to 256 µg/mL and prepared biofilms on silicone elastomer surfaces [43]. Their results showed that these highly concentrated lock solutions were not able to reduce 72 h-old *C. parapsilosis* biofilm metabolic activity by more than about 60% (voriconazole) or 40% (posaconazole). Only one study gave divergent results showing that fluconazole, itraconazole, and voriconazole at concentrations of 1 mg/mL eliminated detectable viability in biofilms (5-day-old) made by *C. albicans*, *C. glabrata* and *C. tropicalis* within 7, 10 and 14 days on polyurethane surfaces, respectively [36]. In this treatment, lock solutions were replaced every 2 days. Fourteen days is a very long time and tested lock periods are usually shorter, which may help to explain the divergent data. In addition, these results may be strain-dependant as authors studied only one strain of each species of *Candida*. To our knowledge, this study from Ko et al. is the only one demonstrating that itraconazole, fluconazole, and voriconazole are generally superior to caspofungin and amphotericin B [36].

Finally, in vitro literature data primarily underscores the inability of available azoles, such as fluconazole, itraconazole, voriconazole and posaconazole to reduce the metabolic activity in *C. albicans* biofilms by more than half, regardless of the tested biofilm maturation status, azole concentration and contact duration. Given the failure of in vitro data, no clinical data exist on azole lock. One in vivo study using a rabbit model of *C. albicans* CRBSI was consistent with in vitro models. This study assessed the efficacy of liposomal amphotericin B (L-AmB) compared to fluconazole lock-therapies [44]. The antifungal solutions were locked in the lumen of each catheter for 8 h per day for 7 days. L-AmB was more efficient in catheter sterilization than fluconazole.

## 3. Amphotericin B Lock Solutions

Available data regarding the efficacy of antifungal lock solutions based on amphotericin B deoxycholate (d-AmB), and lipid formulations of amphotericin B, such as amphotericin B lipid complex (ABLC) and L-AmB will be reported in this part. Öncü evaluated in vitro the capacity of amphotericin B lock solutions (36 µg/mL to 120 µg/mL) to eradicate mature (5-day old) biofilms formed on silicone catheters using a viability approach (CFU method) as previously mentioned for azole testing [38]. Results showed that d-AmB significantly decreased the number of yeasts inside catheters, which were completely sterile at the fifth day. Furthermore, d-AmB efficiency was observed as early as the first day whatever the tested concentration. Similar results were obtained on *C. parapsilosis* biofilms using d-AmB at 75 µg/mL to 250 µg/mL. Ko et al. also investigated in vitro the interest of d-AmB lock solutions in inhibiting mature (5-day old) biofilms [36]. *C. albicans*, *C. glabrata* and *C. tropicalis* species (one strain of each) were investigated and d-AmB minimal inhibitory concentrations for biofilm cells (CFU method, polyurethane surfaces) were rather low: 16 mg/mL, 32 mg/mL and 4 mg/mL, respectively. However, as previously mentioned, these authors reported rather divergent results, and found that tested azole compounds were generally superior to d-AmB in eradication of *Candida* biofilms. Finally, Fiori et al. reported on the in vitro anti-biofilm activity of d-AmB lock solutions against *C. albicans*, *C. glabrata*, *C. tropicalis*, *C. krusei* (20 strains of each) and *C. parapsilosis* (40 strains) assessing the inhibition of sessile yeast metabolism caused by the drug (XTT method) [37]. They reported that the lowest d-AmB concentrations at which 50% decrease in biofilm was observed ranged from 0.5 µg/mL to >32 µg/mL for all tested strains of *C. albicans*, *C. glabrata*, *C. parapsilosis*, and *C. tropicalis*, and from 1 µg/mL to >32 µg/mL for those of *C. krusei*. Actually, the concentration responsible for 50% biofilm inhibition was ≤ 1 µg/mL for 45% (*C. parapsilosis*), 40% (*C. tropicalis*), 30% (*C. albicans* and *C. glabrata*) and only 1% (*C. krusei*) of the studied strains. Furthermore, the concentrations causing 90% decrease were >32 µg/mL for *C. albicans, C. parapsilosis* and *C. krusei* strains, whereas they were 32 µg/mL and 16 µg/mL for *C. tropicalis* and *C. glabrata* strains. Finally, even if d-AmB lock solutions displayed little activity against sessile *Candida* yeasts, these results based on the investigation of numerous strains of each studied *Candida* species clearly demonstrated that this activity was both limited and strain- dependent.

However, some early in vitro experiments demonstrated the anti-biofilm activity of lipid formulations of amphotericin B, such as ABLC, and their significantly higher anti-biofilm activity against *C. albicans* and *C. parapsilosis* compared to d-AmB [6]. Three studies published between 2012 and 2016 studying different *Candida* species focused on the efficiency of L-AMB lock solutions in fighting *Candida* biofilms [27,39,44]. Toulet et al. investigated in vitro the activity of L-AmB solutions at 200 µg/mL and 1000 µg/mL against biofilms of *C. albicans*, *C. parapsilosis* and *C. glabrata* on silicone catheters (2 strains of each species, XTT method) and aged 12 h or 5 days. Their efficacy was analysed depending on lock duration (4 h, 12 h or 24 h) and in each case the authors evaluated post-lock remanence [27]. They showed that L-AmB solutions used at 1000 µg/mL reduced 12 h and 5-day-old *Candida* spp. biofilms regardless of lock duration (inhibition always ≥80% for *C. albicans* and *C. glabrata* and ≥62% for *C. glabrata*), but could never totally eradicate biofilms. Lower activities were obtained using L-AmB at 200 g/mL, with inhibition percentages for *C. albicans*, *C. glabrata* and *C. parapsilosis* ranging from 73.5% to 91.5%, 77.5% to 93% and 41.5% to 88.5%, respectively. This study showed that L-AmB solutions at 1000 µL/mL used for short lock times (≤4 h) could significantly reduce biofilms of *C. albicans* and *C. glabrata* for up to 48 h (post-lock remanence) but were less efficient against *C. parapsilosis* biofilms. Basas et al. also evaluated the efficacy of L-AmB in reducing mature biofilms (48 h-old) of *C. parapsilosis* on silicone surfaces, studying two strains [40]. They reported that the lowest L-AmB concentrations causing a 50% and a 90% decrease in *C. parapsilosis* biofilm were 0.5 or 1 µg/mL (depending on the strain) and >1024 µg/mL, respectively, confirming how difficult it is to totally eradicate biofilms, even if the drug remains effective. Biofilm reduction was evaluated according to decreases in yeast viability (Live/dead staining) and metabolism (XTT method). Interestingly, these authors compared L-AmB activities depending on the biofilm substrate and showed that 48 h-old biofilms formed on polystyrene surfaces were much easier to reduce compared to those prepared on silicone: 50% biofilm reduction at 0.125 µg/mL or 0.25 µg/mL depending on the strain, and 90% reduction at 8 µg/mL or 16 µg/mL. This finding highlights the influence of substrates and the need to use materials mimicking those used to manufacture medical devices. Finally, Simitsopolous et al. investigated in vitro the interest of L-AmB against mature biofilms (48 h-old) of *C. albicans* (1 strain), *C. lusitaniae* (6 strains) and *C. guilliermondii* (5 strains) using both metabolic (XTT) and viability (CFU counts) approaches, considering locks for 24 h [39]. In these conditions, the lowest L-AmB concentrations causing 50% decrease in biofilms were 0.25 µg/mL, 2 µg/mL and 0.125 µg/mL for *C. albicans*, *C. guilliermondii* and *C. lusitaniae*, respectively, which seems close to the results presented by Fiori et al. [37]. At 256 µg/mL to 2048 µg/mL, L-AmB induced strong biofilm damage against *C. albicans* (about 80% to 90% decrease), which is consistent with the results of Toulet et al. [27].

Few patients have been treated with d-AmB antifungal lock [25,45,46,47,48,49,50] (Table 2). d-AmB antifungal lock has been shown to be effective in 6/10 patients. However, failures of therapy are often unpublished. In addition, the patients received different systemic antifungal therapies that were likely to bias analysis of these data [25]. Nevertheless, d-AmB as an antifungal lock failed to find a place in a clinical setting, and was not reinforced by animal models. Indeed, the only experimental in vivo model comparing d-AmB and caspofungin was favoured of caspofungin [51]. Shuford et al. used a rabbit model of *C. albicans* catheter-related bloodstream infection (CRBSI). A silicone catheter was inserted into the external jugular vein of the rabbit. The infected catheter was used daily to administered an antifungal drug and was locked after injection with a solution containing heparin and the antifungal drug. Using d-AmB, 13/16 catheters were sterile at day 7 compared to all catheters locked with caspofungin [51].

The other in vivo models focused on lipid forms of Amphotericin B. Mukherjee et al. used a rabbit model of *C. albicans* CRBSI to test antifungal lock with ABLC [52]. A silicone catheter was locked for 4 or 8 h per day with a solution containing ABLC and heparinized saline. At day 11 of antifungal lock, ABLC-locked catheters were all sterilized compared to control, whatever the duration of lock therapy. In another model, L-AmB was superior to fluconazole in eradicating *C. albicans* biofilm [44]. All these animal studies used *C. albicans* and silicone catheters.

L-AmB has been used as lock therapy for few patients [53,54,55] (Table 2). L-AmB lock was used in a patient on hemodialysis with persistent CRBSI due to *C. albicans* on a polyurethane catheter [53]. The catheter could not be removed due to lack of other available sites for vascular access. It was exchanged over a wire. A lock solution containing 2.67 mg/mL of L-AmB in 5% dextrose plus 200 UI (66 UI/mL) of heparin was prepared and dwelled for 12 h in the lumen of the new catheter. Antifungal lock with L-AmB was continued for 6 days concomitantly with systemic micafungin. Systemic antifungal treatment was prolonged 6 months without any relapse of fungal infection. The same protocol of L-AmB lock was used for the other cases, leading to success in 7/8 episodes [54,55].

The only clinical prospective study of antifungal lock came from a pediatric context [56]. This study involved L-AmB and various polyurethane and silicone central venous catheters (CVC) from different manufacturers. L-AmB solution at 2 mg/mL was instilled daily into the catheter in 12 children having 13 episodes of *Candida* CRBSI [56]. The antifungal solution dwelled for 8–12 h in the catheter lumen. All children received an additional systemic antifungal drug, either caspofungin or L-AmB. Blood cultures from each line of the CVC were collected every day. The species involved were *C. albicans*, *C. glabrata*, *C. lusitaniae*, *C. parapsilosis*, and *Rhodotorula rubra*. The primary endpoint was two consecutive negative CVC-bloodcultures within 5 days of initiating antifungal lock. A total of 10 infections in 10 patients (77%) met the primary endpoint, and cure without relapse was obtained in 5 of 13 (38%) infections. Culture became negative within 2.4 days (range 1–5 days). Although the number of patients of this pilot study was insufficient to conclude that L-AmB antifungal lock was fully effective, it provided encouraging results.

Finally, available data obtained with in vitro models generally suggest better activity of L-AmB lock solutions compared to d-AmB lock-solutions; they suggest that L-AmB lock solutions could help to significantly reduce *Candida* spp. mature biofilms without being able to totally eradicate sessile yeasts. They highlight the influence of both the nature of the substrate (polystyrene, silicone, polyurethane, and so on), lock duration, and age of the treated biofilm.

## 4. Echinocandin Lock Solutions

Echinocandins act by inhibiting the biosynthesis of 1.3-beta-glucans, which are key constituents of the fungal cell wall, and this mode of action has encouraged researchers to investigate their anti-biofilm activity. The anti-adherent and anti-biofilm interest of echinocandins was rapidly shown, even using low concentrations, close to MIC [6,9]. More recently, a study done on more than 200 *Candida* isolates helped to show that whereas fluconazole belonged to non-active anti-biofilm agents, caspofungin belonged to highly active anti-biofilm agents [24]. Anidulafungin, caspofungin and micafungin all belong to echinocandins, and yet most studies investigating the echinocandin possibly used in lock therapy focus on caspofungin, and to a lesser extent on micafungin and anidulafungin.

Cateau et al. have developed an in vitro model to evaluate the effect of a 12 h lock with caspofungin (2 µg/mL) or micafungin (5 µg/mL) solutions against young (12 h old) or mature (5 days old) *C. albicans* on silicone surfaces; tested concentrations corresponded to about 100 times MIC and anti-biofilm activity was evaluated based on reduction in the metabolic activity of sessile yeasts (XTT method) [35]. Their results showed that even 48 h after the end of the lock, 12 h and 5-day-old biofilms treated by caspofungin and micafungin were still inhibitedby at least 55% (caspofungin: ≈60% to ≈90%) (micafungin: ≈55% to ≈92%) and 47% (caspofungin: ≈47% to ≈88%) (micafungin: ≈54% to ≈91%), respectively. The weakness of this study is that only two *C. albicans* strains were studied and that they were collection instead of clinical strains. However, the same lock-model was then applied to biofilms prepared in the same manner, with all 8 *C. albicans* and 6 *C. glabrata* clinical strains isolated from infected catheters, and this confirmed the interest of echinocandin lock-solutions [26]. *C. glabrata* clinical strains were all isolated from infected catheters, and thereby confirmed the interest of echinocandin lock-solutions. Irrespective of antifungal concentration and biofilm age (12 h or 5 days old), echinocandins still inhibited *C. albicans* biofilms by at least 65% (caspofungin: inhibition ≥≈77 %, micafungin: ≥≈65%) 48 h after the end of the lock. The strain dependence of results was mentioned in the case of mature 5-day-old biofilm but not for 12 h ones. Regarding *C. glabrata* biofilms, inhibitory percentages caused by caspofungin at 5 g/mL were still at least 55.8% (12 h biofilms) or 44.4% (5 days old) 48 h after the end of the lock compared to those of micafungin at 5 g/mL which were still at least 92.4% (12 h old biofilms) or 90.3% (5 days old). So, micafungin showed greater sustained efficacy than caspofungin against both 12 h and 5-day-old biofilms of *C. glabrata*, whereas there was no obvious difference in the case of *C. albicans* biofilms. In any event, none of the tested conditions totally eradicated biofilms, as was reported by the same team regarding L-AmB [27]. Öncü studied the killing activity (CFU counts) of caspofungin solutions (300 to 1000 times MIC) against 5-day-old biofilms on silicone catheters; the lock periods were 1, 3, 5 and 7 days, caspofungin solutions being replaced every 2 days) [38]. The weakness of this study is that only two strains were studied, one from each species. The results showed that, for both *C. albicans* and *C. parapsilosis* biofilms, caspofungin lock solutions significantly decreased the number of yeasts inside catheters, starting on the first day of the treatment, and the catheters were completely sterile at the fifth day, which corresponds to efficacy comparable to that reported by this author using amphotericin B, as mentioned in the previous part.

Unlike others, Ko et al. reported quite low activity (CFU method) of caspofungin lock solutions against 5-day-old biofilms of *C. albicans* and *C. glabrata* on polyurethane surfaces, the lowest caspofungin concentrations at which a 50% decrease in biofilm was observed remaining >256 µg/mL [36].

Only the study of Simitsopoulou et al. compared the activity on anidulafungin, caspofungin and micafungin lock solutions for 24 h against metabolism and cultivability of sessile yeasts from mature (48 h old) biofilms of *C. albicans*; however, they considered only one collection strain [39]. The lowest average concentrations at which a 50% decrease in *C. albicans* biofilm was observed were very close to each other, whatever the tested drug: 0.25 µg/mL for both micafungin and anidulafungin and 0.5 µg/mL for caspofungin. However, the highest inhibition of sessile yeast metabolism reached 100% and was only obtained by caspofungin, the other echinocandins being less active. This was similar to results reported by the team using L-AmB, as mentioned in the previous part; taken as a whole these results suggested the high and comparable in vitro efficacy of L-AmB and caspofungin against mature biofilms of *C. albicans*. Simitsopoulou et al. extended the study to 6 and 5 clinical strains of *C. lusitaniae* and *C. guilliermondii*, respectively and showed that anidulafungin and micafungin had reduced activity against *C. lusitaniae* and *C. guilliermondii* biofilms compared with caspofungin: the lowest concentration of anidulafungin and micafungin at which a 50% decrease in biofilm was observed ranged from 32 µg/mL to >2,048 µg/mL, biofilms of *C. lusitaniae* being the most resistant [39]. Interestingly, caspofungin was the most active agent against *C. lusitaniae* and *C. guilliermondii* biofilms, achieving complete and persistent (up to 72 h post-lock) biofilm eradication at lock concentrations ranging from between 512 µg/mL to 2048 µg/mL depending on the tested strains. Fiori et al. compared the anti-biofilm activity of anidulafungin and caspofungin lock solutions in the presence or absence of 50% serum, studying numerous clinical strains of 5 *Candida* species [37]. Overall, the presence of serum increased concentrations at which a 50% decrease in biofilm was observed, whatever the tested echinocandin, and these concentrations were generally lower for anidulafungin compared to caspofungin, suggesting the superiority of anidulafungin against sessile yeasts. More precisely, in presence of serum, concentrations were relatively close for *C. albicans* and *C. tropicalis* biofilms, ranging from 0.5 µg/mL to 2 µg/mL (anidulafungin) or 2 µg/mL to 8 µg/mL (caspofungin) and 1 µg/mL to 4 µg/mL (anidulafungin) or 2 µg/mL to 8 µg/mL (caspofungin), respectively. Anidulafungin was clearly more active against *C. glabrata* (between 1 µg/mL and 2 µg/mL (anidulafungin) or between 2 µg/mL and 16 µg/mL (caspofungin)) and *C. krusei* (between 4 µg/mL and 16 µg/mL (anidulafungin) or ≥8 µg/mL (caspofungin)). However, both were only weakly active against *C. parapsilosis* (≥4 µg/mL). Finally, the results of Basas et al. were based on metabolic (XTT method) and viability tests (staining) and underscored the high efficacy of anidulafungin against *C. parapsilosis* mature biofilms (48 h-old) on silicone surfaces: they reported a concentration at which a 50% or 90% decrease in biofilm was observed ≤0.25 µg/mL or equal to 1 µg/mL. Under the test conditions, anidulafungin thus appeared much more effective than L-AmB [40]. Lown et al. recently studied the efficacy of a 24 h lock treatment based on micafungin used alone or combined with ethanol and/or doxycycline in eradicating a 24 h-old biofilm developed in polystyrene microplate wells under static conditions [41]. Five *C. albicans* strains were studied, two clinical ones, derived echinocandin-resistant isolates and three collection ones, and anti-biofilm activity was evaluated with metabolic (XTT) and cultivability (CFU counts) assays. The results demonstrated that the combination comprising 20% ethanol, 0.01565 µg/mL micafungin and 800 g/mL doxycycline was highly effective against both forming and mature *C. albicans*. This combined lock-solution reduced the metabolic activity of treated biofilms to ≤2%, and prevented the regrowth from both forming and mature biofilms; however, this solution was not more active than 20% ethanol used alone. By using a three-drug lock therapy approach, authors wished to maximize the broad-spectrum activity of the solution to include both yeasts and bacteria, which would also probably reduce the risk for resistance development.

Lown et al. have mentioned that only limited investigations have been performed on the effect of high concentrations of ethanol on catheter integrity, depending on the material. Use of such a complex three-drug lock solution may consequently help to minimize the ethanol concentration used. An approach combining a 1:1 mixture of 70% ethanol and micafungin 5 mg/L was applied as antifungal lock therapy to treat a preterm infant for *C. albicans* CRBSI [57]. The patient also received systemic L-AmB at 5 mg/kg/day. The lock solution dwelled for 12 h. After 48 h a combination therapy of L-AmB and micafungin for persistent positive blood cultures was administered, and the catheter was locked again for another 12 h with success. Systemic antifungal therapy was discontinued after 21 days. The stability of the lock mixture was tested using high-performance liquid chromatography dosages. The solution of micafungin plus ethanol was stable for at least 24 h.

Caspofungin lock was likewise effective against *C. albicans* CRBSI after 24 h in a mouse model of catheter infection [58]. Catheters recovered from caspofungin pre-treated animals had significantly less biofilm formation compared with untreated controls. There was also a significant decrease in dissemination to kidneys with caspofungin. In the clinical setting, Isguder et al. reported a case of *C. parapsilosis* antifungal lock failure with caspofungin [59] (Table 2). The antifungal lock was started 7 days after the systemic caspofungin and failed to sterilize the CVC. The solution was at a concentration of 3.3 mg/mL with 200 UI of heparin. The authors underlined that optimal concentration of caspofungin lock has not yet been determined. However, another team reported the success of caspofungin lock with the same antifungal lock protocol in a 9-year-old child for *C. lipolytica* CRBSI [60]. The antifungal lock was instilled on the same day as systemic antifungal drug administration. Since the formation of *Candida* biofilm on catheter is known to occur within 24 h, the timing of antifungal lock instillation in the catheter may be crucial to success [61].

Antifungal lock with echinocandins seems to be more effective than that with L-AmB, at least in animal models, as no comparative clinical data are available [40]. A silicone CVC was inserted into New Zealand white male rabbits’ jugular vein, and then filled with *C. parapsilosis*. A lock containing anidulafungin at 3.3 mg/mL or L-AmB at 5.5 mg/mL supplemented with 100 UI of sodium heparin/mL was carried out. After 48 h of antifungal lock, anidulafungin achieved a significant reduction of fungal load and a significant percentage of negative catheter cultures compared to L-AmB. No data were provided concerning the stability of antifungal solution that dwelled for 48 h. Stability and compatibility of antifungal drugs with other solutions has yet to be extensively studied [62]. It has been reported that caspofungin acetate may be precipitated in a solution with heparin. This phenomenon was concentration-dependent with EDTA [62]. Micafungin seemed physically stable for 24 h in combination with heparin sodium at ambient room temperature, as did ABLC with EDTA for at least 8 h [62,63].

## 5. Conclusions

The best antifungal lock solution has to be active, whatever be the catheter material. It has to eradicate biofilm in a timely manner, dwelling less than 12 h in order to allow the patient to continue another systemic treatment or parenteral nutrition. It has to remain stable with anticoagulant agents being used to avoid catheter thrombosis. The anti-biofilm activity of lock solutions based on echinocandins and lipid formulations of amphotericin B has been widely investigated in vitro and their efficacy has been convincingly demonstrated. However, the experimental conditions used by authors are too variable to draw any conclusion as to which of the solutions are the most active. However, the presently available in vitro results suggest the promise of lipid formulations of amphotericin B and echinocandins used as lock solutions to maintain catheters in patients when their removal is not possible. Importantly, according to the reported studies total eradication of sessile yeasts is rarely achieved, and when it is, obtained, usually the described conditions do not seem compatible with a lock therapy, especially on account of long lock duration (often at least 24 h). To conclude, caspofungin, micafungin, anidulafungin and L-AmB are good candidates for lock therapy in further clinical studies, but have imperatively to be combined with systemic therapy. On this subject, the interest of combining a lipid amphotericin B formulation and an echinocandin, one for lock-treatment and the other for systemic treatment, needs to be further investigated and evaluated, while bearing in mind that approach could effectively combat *Candida* spp. biofilms. In addition, further in vivo studies should be focused on the different materials used in the daily clinical practice (polyurethane, silicone).

## Figures and Tables

**Table 1:** Methods to evaluate the activity of antifungal lock solutions according to selected in vitro studies. d-AmB = Amphotericin B deoxycholate; L-AmB = liposomal Amphotericin B; ALT: antifungal lock treatment; XTT: 2,3-bis-(2-methoxy-4-nitro-5-sulfophenyl)-2*H*-tetrazolium-5-carboxanilide.

*Candida* Species and Number of Strains	Age of the Treated Biofilm	Surface Nature	Antifungal Solutions	ALT Duration	Investigation of Treatment Persistence	Method for Antibiofilm Evaluation	Major Conclusions	Reference
*C. albicans* (2)	12 h and 5 days	100% silicone	Caspofungin; micafungin	12 h	24, 48 and 72 h post-lock	XTT	48 h-lock of caspofungin at 2 µg/mL or micafungin at 5 µg/mL reduced biofilms by 47%	Cateau et al., 2008 [35]
*C. albicans* (1); *C. glabrata* (1); *C. tropicalis* (1)	5 days (with shaking)	polyurethane	d-AmB; fluconazole; itraconazole; voriconazole; caspofungin	1, 3, 5, 7, 10 and 14 days (lock solutions replaced every 2 days)	none	XTT + CFU counts	Azoles at 1 mg/mL eradicated all biofilms within 7 to 14 days; azoles were superior to d-AmB and caspofungin to eradicate biofilms	Ko et al., 2010 [36]
*C. albicans* (10); *C. glabrata* (6)	12 h and 5 days	100% silicone	Posaconazole; Caspofungin; micafungin	12 h	24, 48 and 72 h post-lock	XTT	48 h-lock of posaconazole reduced *C. albicans* biofilms by <50% compared to ≥60% with echinocandins. Greater sustained efficacy of micafungin compared with caspofungin against all biofilms of *C. glabrata*, albeit no obvious difference for *C. albicans* biofilms	Cateau et al., 2011 [26]
*C. albicans*(20); *C. parapsilosis* (40); *C. tropicalis* (20); *C. glabrata* (20); *C. krusei* (20)	24 h	polystyrene	d-AmB; voriconazole; anidulafungin; caspofungin	24 h	none	XTT	inefficacy of azoles against all species of *C. albicans* biofilms; d-AmB activity was limited and strain dependent. Anidulafungin more active than caspofungin	Fiori et al., 2011 [37]
*C. albicans* (1); *C. parapsilosis* (1)	5 days	silicone	d-AmB; fluconazole; itraconazole; voriconazole; caspofungin	1, 3, 5, 7 days (lock solutions replaced every 2 days)	none	Determination of viable count (no details)	No azole can sterilize catheters; catheters treated with d-AmB or caspofungin were completely sterile at the fifth day	Öncü et al., 2011 [38]
*C. albicans* (2); *C. glabrata* (2); *C. parapsilosis* (2)	12 h and 5 days	100% silicone	L-AmB;	4, 12 and 24 h	24 and 48 h	XTT	High and persistent inhibitory activity of L-AmB used at 1000 µg/mL for short lock but no full biofilm eradication; *C. parapsilosis* biofilm less susceptible than that of other species.	Toulet et al., 2012 [27]
*C. albicans* (1); *C. lusitaniae* (6); *C. guillermondii* (5)	48 h	polystyrene	L-AmB; anidulafungin; caspofungin; micafungin	24 h	24, 48 and 72 h post-lock	XTT + CFU counts	L-AmB at 256 to 2048 µg/mL inhibited 80 to 90% of *C. albicans* biofilm; comparable in vitro efficacy of L-AmB and caspofungin against *C. albicans* mature biofilms; anidulafungin and micafungin less active than caspofungin against *C. albicans*, *C. guilliermondii* and *C. lusitaniae*.	Simitsopoulou et al., 2014 [39]
*C. parapsilosis* (2)	48 h	silicone; polystyrene	L-AmB; anidulafungin	48 h	none	XTT	50% biofilm reduction was obtained with 4-fold less anidulafungin (≤0.25 µg/mL) than L-AmB (1 µg/mL); 90% biofilm reduction was obtained using anidulafungin at 1 µg/mL compared to L-AmB at >1024 µg/mL	Basas et al., 2016 [40]
*C. albicans* (5)	24 h	Silicone; polystyrene	micafungin	24 h	none	XTT and CFU counts	High efficacy of the combination: 20% ethanol, 0.01565 µg/mL micafungin and 800 g/mL doxycycline against forming and mature biofilms	Lown et al., 2016 [41]

**Table 2:** Antifungal lock in clinical setting. Only cases with sufficient data were included in the table. For all cases, see ref [25]. There is no data for azole antifungal lock or other type of echinocandins. d-AmB = Amphotericin B deoxycholate; L-AmB = liposomal Amphotericin B; ALT: antifungal lock treatment; BC: blood culture.

Pient Age	Episode Number	Systemic Antifungal Treatment	ALT Duration	Antifungal Lock Solution	Fungus	Major Conclusion	Reference
deoxycholate AmB							
35 y.o.	1	No systemic therapy	12 h/day for 21 days	d-AmB 2.5 mg/mL	Malassezia furfur	Success; BC negative after 7 days of ALT; fever resolution after 2 days of ALT	Arnow et al. [46]
30 y.o.	1	d-AmB for 3 days then fluconazole for 4 days	8–12 h/day for 15 days	d-AmB 2.5 mg/mL	*C. glabrata*	Initial success but relapse 6 weeks later; no details on BC	Benoit et al. [47]
40 y.o.	1	d-Amb for 1 day	6 h/day for 7 months	d-AmB 2.5 mg/mL	*C. albicans* + *C. glabrata*	Eradiaction of *C. albicans* but failure for *C. glabrata* that recurred 5 days after ALT discontinuation	
	2	Fluconazole for 3 days	6 h/day for 8 months	d-AmB 2.5 mg/mL	*C. glabrata*	Successafter 8 months of ATL; BC negative for 8 months; no data after ALT withdrawal	
13 y.o.	1	d-AmB for 6 days then fluconazole (no duration)	24 h/day for 20 days	d-AmB 2.5 mg/mL	*C. parapsilosis*	Success; ALT started after 6 days of systemic antifungal therapy;	Wu et al. [48]
2 y.o.	1	d-AmB for 7 days	12 h/day for 14 days	d-AmB 2.5 mg/mL	*C. albicans*	Success; fever resolution after 3 days of ALT; BC negative at the end of ALT	Viale et al. [49]
65 y.o.	1	Fluconazole for 7 days	12 h/day for 14 days	d-AmB 2.5 mg/mL	*C. albicans*	Success; fever resolution after 4 days of ALT; BC negative at the end of ALT	
40 y.o.	1	Fluconazole (no duration)	6 h/day for 14 days	d-AmB 5 mg/mL	*C. glabrata*	Success; ALT started 2 days after antifungal systemic; surveillance BC negative; no further details	Angel-Moreno et al. [50]
liposomal AmB							
infant	1	L-AmB for 14 days	8 h/day for 14 days	2.67 mg/mL + heparin 100 UI	*C. parapsilosis*	Success; BC negative after 2 days of ALT	Castagnola et al. [53]
17 month-old	1	L-AmB for 8 days	8 h/day for 7 days	2.67 mg/mL + heparin 200 UI	*C. glabrata* + *C. albicans*	Success; ALT started at 24 h of systemic treatment	Buckler et al. [54]
	2	L-AmB for 10 days	8 h/day for 10 days	2.67 mg/mL + heparin 200 UI	*C. albicans*+ *C. glabrata*	Failure; no details	
	3	L-AmB for 16 days	8 h/day for 16 days	2.67 mg/mL + heparin 200 UI	*C. glabrata*	Success; BC negative after 6 days of ALT	
7 y.o.	1	L-AmB for 21 days	8 h/day for 17 days	2.67 mg/mL + heparin 200 UI	*C. albicans*	Success; ALT started at 24 h; BC negative after 24 h of ALT	
6 month-old	1	L-AmB for 15 days	8 h/day for 15 days	2.67 mg/mL + heparin 200 UI	*C. parapsilosis*	Success; BC negative after 8 days of ALT	
1 y.o.	1	L-AmB for 14 days	8 h/day for 14 days	2.67 mg/mL + heparin 200 UI	*C. guillermondii*	Success; BC negative after 3 days of ALT	
64 y.o.	1	Micafungin for 14 days	24 h/day, change every 12 h, for 6 days	2.67 mg/mL + heparin 200 UI	*C. albicans*	Success; BC negative before initiation of ALT; ALT started after catheter exchange over a wire and after 9 days of systemic antifungal	Paul Dimondi et al. [55]
Caspofungin							
9 y.o.	1	caspofungin	12 h/day for 14 days	3.33 mg/mL + heparin 200 UI	C. lipolytica	Success; BC negative after 4 days of ALT	Özdemir et al. [52]
1.5 y.o.	1	caspofungin	12 h/day for 14 days	3.33 mg/mL + heparin 200 UI	*C. parapsilosis*	Failure; ALT started 7 days after systemic caspofungin: BC still positive at 14 days	Isgüder et al. [51]
